# Evaluation of a new instrument offering sodium, potassium, and ionized calcium in combination with haematocrit, pH and blood gas analysis; the performance of the Nova Stat Profile 1

**DOI:** 10.1155/S1463924689000040

**Published:** 1989

**Authors:** Susan Schonfield, Peter Frost, Robert Cramb, Chris Royle

**Affiliations:** Department of Chemical Pathology Central Middlesex Hospital Acton Lane London NWIO 7NS UK

## Abstract

The Nova Stat Profile 1 analyser, a combined sodium, potassium, ionized calcium, haematocrit and conventional blood gas analyser, was evaluated over a four month period. In addition to assessing and demonstrating that the instrument met analytical requirements, an appreciation of the use of ionised calcium (iCa) was made. Prospective costs were characterised and practical problems of iCa measurement addressed.


Journal of Automatic Chemistry Vol. 11, No. 1 (January–February 1989), pp. 22–27

### Evaluation of a new instrument offering sodium, potassium, and ionized calcium in combination with haematocrit, pH and blood gas analysis; the performance of the Nova Stat Profile 1

**Susan Schonfield, Peter Frost, Robert Cramb and Chris Royle**

*Department of Chemical Pathology, Central Middlesex Hospital, Acton Lane, London NW10 7NS*

*The Nova Stat Profile 1 analyser, a combined sodium, potassium, ionized calcium, haematocrit and conventional blood gas analyser, was evaluated over a four month period. In addition to assessing and demonstrating that the instrument met analytical requirements, an appreciation of the use of ionised calcium (iCa) was made. Prospective costs were characterised and practical problems of iCa measurement addressed.*

The performance of the Nova Stat Profile 1 (NSP1) was evaluated over a period of four months. The instrument measures pH, $pCO_2$, $pO_2$, haematocrit (hct), barometric pressure. Calculated parameters include total $CO_2$, actual bicarbonate, oxygen content, standard bicarbonate, base excess (ecf/blood), haemoglobin, oxygen saturation and normalized ionized calcium.

There is particular interest in the measurement of iCa as a clinically valuable addition in determining calcium status correctly [1]. Standard methods of automated analysis of total calcium (tCa) use dye-binding techniques, and have been shown to have limitations as a discriminant in patients with disorders of calcium homeostasis [2]. The inclusion of iCa measurement with blood gas analysis offers a potential solution to the introduction of iCa measurement, as it combines similarity of specimen requirements for both tests [3]. Evaluation of the NSP1 provided the opportunity to investigate aspects of iCa measurement, such as sample handling and the effect of the ratio of anticoagulant to sample [4]. The usefulness of iCa measurement in conjunction with blood gas, hct and other electrolyte measurement was also reviewed. Projected costs were characterized for varying workloads.

### Description of the instrument

The NSP1 is shown in figure 1. Its dimensions are 56.3 cm deep by 56.3 cm wide by 46.1 cm high. It weighs 45 kg and has three major component areas: (1) a microprocessor, screen and keyboard; (2) a centrally placed fluids deck with reagent pack, and below it, the printer unit; and (3) the analytical compartment, which contains the electrodes, the barometric pressure module and temperature regulation device. The electrodes are of the flow-by design, and are arranged in the following order:

| | |
|---|---|
| Reference electrode | solid state electrode |
| $Na^+$ | sodium ion-selective electrode glass |
| $K^+$ | valinomycin ion-exchange membrane |
| iCa | neutral carrier membrane electrode |
| pH | hydrogen ion-selective electrode glass |
| $pCO_2$ | Severinghaus-type electrode |
| $pO_2$ | polarographic Clark-type electrode |
| hct | impedance electrode. |

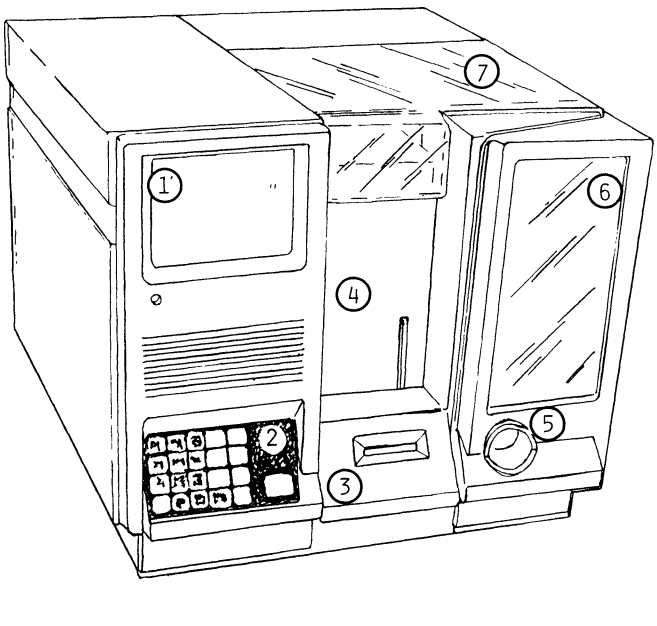

1 CRT Display
2 Keypad
3 Printer
4 Reagent Pack
5 Sampling Inlet
6 Analytical Compartment
7 Fluids Deck

*Figure 1. Nova Stat Profile 1.*

Correspondence to Dr Peter Frost.

0142–0453/89 $3.00 © 1989 Taylor & Francis Ltd.

Any determination may be made on its own or in 'mini profile' groups. Sample type may be venous or arterial whole blood, plasma, serum or expired gases.

### Materials and methods

The NSP1 was supplied by the manufacturers (Nova Biomedical, Corsham, UK) and installed by the U.K. agent (Clandon Scientific Ltd, Aldershot, Hampshire, UK) who also provided a back-up service. The NSP1 was operated throughout according to the manufacturer's instructions. Manufacturer's control materials, both aqueous and protein-based, were used to evaluate within- and between-day imprecision and bias.

Within-day imprecision was measured by 20 replicate analyses of the sodium, potassium, iCa and pH, $pCO_2$ and $pO_2$ at three levels on the aqueous control material; sodium, potassium and iCa were also analysed by 20 replicate analyses at three levels on the protein-based control material. Hct was measured by 20 replicate analyses on the manufacturer's control material at two levels. The manufacturer's instructions were strictly followed – aqueous material was shaken for 10 s, opened and used within 60 s. Protein-based material was gently mixed, opened and used within 48 h, having been stored at 4 °C during this time.

Between-day imprecision was evaluated by measuring controls (as described above) in triplicate daily for 20 days.

Bias was evaluated with similar materials as those used for within- and between-day imprecision. Between-day imprecision and bias for blood gases were also evaluated by thin film tonometry, using an IL237 tonometer (Instrumentation Laboratories, Warrington, UK). This instrument was used and maintained as recommended by the manufacturers. Blood gases at two levels (high and low) were measured in triplicate for 20 days. Concentrations of gases at the high level were $pCO_2$ 13.5%, $pO_2$ 13.9% of the total (remainder nitrogen), and at the low level were $pCO_2$ 7.32%, $pO_2$ 6.8% of the total (remainder nitrogen). Sample preparation was standardized as follows. 5 ml of whole blood from a lithium heparin collection tube was added to the sample cuvette and placed in the tonometer for at least 5 min to attain temperature equilibration. The equilibrating gas was bubbled through the tonometer at a flow rate of 300 ml/min to saturate the chamber. Whole blood was then tonometered for 10 min to ensure complete saturation at the same gas flow rate. A 1 ml syringe (Insulin type) was used to sample the blood. Sample transfer to the NSP1 was minimized by priming the instrument to sample immediately after withdrawal from the tonometer.

While the sample was being measured on the NSP1, tonometry was continued on the IL237, until three samples had been measured for each gas level. For each observed result the theoretical result was calculated for that day's barometric pressure, and the observed result was then corrected to standard barometric pressure (760 mmHg). Between-day imprecision and bias were then obtained from these corrected values.

### Patient sample comparison

*Correlation with existing methods*

*Blood gases*

61 patient arterial whole blood samples collected into Pulsator syringes (Concord Laboratories Ltd, Folkstone, UK, sodium heparin content 150 U.S.P. units) were assayed for pH, $pCO_2$ and $pO_2$, firstly on a Corning 168 (Corning Medical & Scientific, Halstead, UK) and then, in all cases within 2 min, on the NSP1.

*Haematocrit*

148 patient samples collected into $Na_2$ E.D.T.A. containers were assayed, over a period of five consecutive days. The samples were selected from those run on the previous day on a Technicon H6000 (Technicon, Basingstoke, UK), to cover as wide a range of values as possible.

*Sodium and potassium*

197 patient samples of plasma obtained from whole blood collected into lithium heparin containers were assayed for sodium and potassium on the Beckman Astra 4 (Beckman, High Wycombe, UK). 150 were then assayed on the same day on the NSP1. 47 samples were selected since the $Na^+$ and/or $K^+$ values were outside the appropriate reference range (sodium 135–145 mmol/l and potassium 3·5–5·0 mmol/l). These samples were frozen and stored at −20 °C; when thawed they were mixed gently, centrifuged for 5 min at an RCF of 850 g (Beckman Tabletop centrifuge TH4 rotor with buckets). They were then decanted into sample cups, reassayed for sodium and potassium on the Astra 4 and immediately thereafter, on the NSP1.

*Statistical analysis*

Linear regression analysis by the method of Deming was performed on the data obtained from comparative analyses [5].

### Ionized calcium

*Length of time prior to assay*

Blood for serum and plasma samples, taken consecutively without stasis were either placed in chilled water, centrifuged for 5 min as above, at 4 °C and analysed immediately, or were left for different time intervals and then centrifuged as above prior to analysis. All sample bottles were filled to within 1 cm of the top to minimize the effect of carbon dioxide loss [6]. iCa values were corrected for a pH of 7.40 (NSP1 printout as '$nCa^{++}$'). These specimens were then analysed for tCa on the ERIS analyser (BDH, Poole, UK), using an o-cresol-phthalein-complexone method (ERIS kit, BDH), coefficient of variation 2.5%.

*Effect of anticoagulant*

Different sample volumes of venous blood were taken in Pulsator syringes from which all the visible sodium heparin solution had been expelled. 150 µl remain in the 'dead space' – i.e. 150 U.S.P. units of heparin sodium

(Concord Laboratories Ltd). iCa, sodium and potassium were measured on these samples.

*Patient samples*

Samples were analysed for iCa and tCa (method as above) from 14 patients who were hypercalcaemic (reference ranges: iCa 0·80–1·20 mmol/l, tCa 2·20–2·60 mmol/l). The samples were either arterial whole blood or plasma. The latter was from blood that had been taken into lithium heparin containers filled to capacity, centrifuged and measured within 2 h. iCa values were corrected to a pH of 7·40 for comparison.

*Costs*

Running costs during the evaluation were divided by the number of samples analysed. Projected basic costs for running the instrument per annum outside warranty, for maintenance only, were calculated. Maintenance costs were calculated from manufacturer's schedule for preventive maintenance and include conditioning and cleaning solutions, electrolyte solutions, replacement harness tubing, membraning kits and fan filter replacements. Prices were itemized from the list supplied by the UK distributor in January 1987.

*User appraisal*

A full appraisal of the machine was made. A record of daily electrode slopes was kept and daily maintenance of the machine was performed as recommended by the manufacturers. A variety of staff (MLSO and junior medical) were taught how to use the instrument. The machine had to be re-sited three times during the evaluation due to relocation of the department at that time.

### Results

*Imprecision*

The results are shown in table 1. Between-day coefficients of variation (CVs) are represented by the range of results observed across three control levels.

*Bias*

*Within-day*: using the manufacturer's control material, all parameters were within the target values for the materials at all levels for both aqueous and protein-based standards.

*Between-day*: all parameter means were within the target values for all materials at all levels except for pH on aqueous material (level 2). Later information from the manufacturer indicated that this was not an isolated observation, and that other users had found a consistently higher value than the quoted target. Between-day bias, calculated from tonometry data is also shown in table 1.

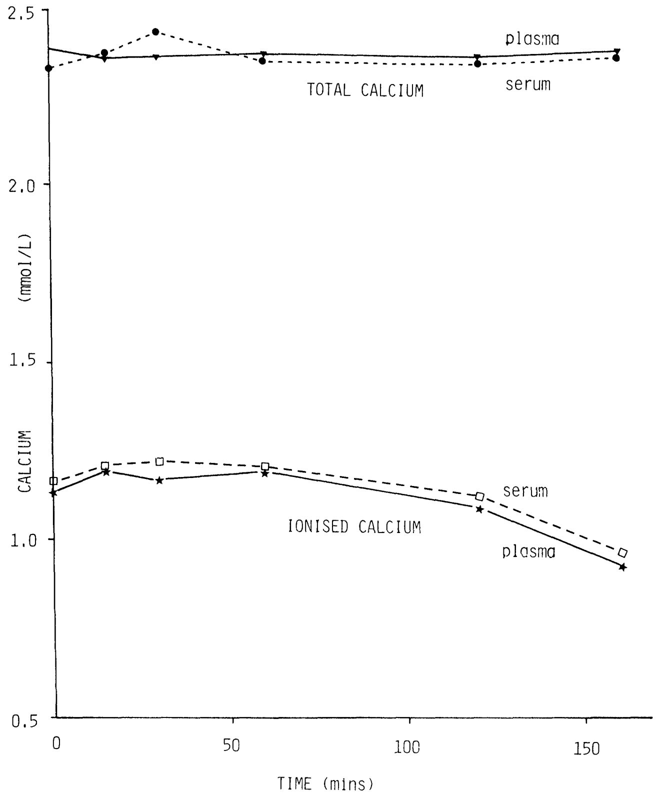

*Figure 2. Effect of length of time prior to assay on ionized calcium.*

*Table 1. Imprecision. (Manufacturer's control material; observed range at three control levels).*

| Analyte | Within-day level 1 | 2 | 3 | | Between-day level 1 | 2 | 3 |
|---|---|---|---|---|---|---|---|
| pH (s.d.) | 0·002 | 0·004 | 0·004 | | 0·010 | 0·010 | 0·011 |
| | | | | CV% | | | |
| $pCO_2$ | 1·01 | 1·07 | 1·87 | | 4·8 | 4·4 | 5·3 |
| $pO_2$ | 2·54 | 0·82 | 1·05 | | 3·1 | 2·0 | 2·9 |
| $Na^+$ | 0·28 | 0·44 | 0·50 | | 0·8 | 0·7 | 0·7 |
| $K^+$ | 0·71 | 0·70 | 1·14 | | 0·9 | 1·0 | 1·3 |
| iCa | 0·78 | 2·30 | 2·53 | | 2·6 | 2·0 | 3·5 |
| hct | 1·59 | 0·99 | — | | 3·3 | 1·0 | — |

TONOMETRY (n = 60)

| | between-day CV % Low gas | High gas | bias (mm Hg) Low gas Actual | Low gas Observed mean | High gas Actual | High gas Observed mean |
|---|---|---|---|---|---|---|
| $pCO_2$ | 4·3 | 4·1 | 52·19 | 52·95 | 96·26 | 97·34 |
| $pO_2$ | 4·9 | 1·7 | 48·48 | 47·76 | 99·11 | 96·23 |

*Table 2. Patient sample comparison. ($y = A + Bx$, where $y$ = NSP1 method, $x$ as below).*

| | A | B | R | X | R quoted by Nova* |
|---|---|---|---|---|---|
| pH | −0·1474 | 1·0207 | 0·931 | Corning 168 | 0·993 |
| $pCO_2$ | −0·8043 | 1·0805 | 0·973 | ,, | 0·998 |
| $pO_2$ | 2·6136 | 1·025 | 0·996 | ,, | 0·997 |
| $Na^+$ | 3·2119 | 0·997 | 0·948 | Beckman Astra 4 | 0·991 |
| $K^+$ | −0·0511 | 1·045 | 0·990 | ,, | 1·006** |
| hct | 0·5649 | 0·9444 | 0·902 | Technicon H6000 | 0·998 |

* From *The Future of Blood Gas* (Nova leaflet). Comparison methods not quoted for $Na^+$, $K^+$ and hct. Nova comparison instrument for blood gases (Corning 178).
** This figure as published by Nova.

*Calcium*

Effect of length of time prior to assay iCa levels fell markedly after one hour (see figure 2). There was no significant difference in tCa levels.

*Anticoagulant effect*

iCa levels decreased as anticoagulant to sample proportion increased. There was a similar, smaller effect on the potassium levels (see figure 3).

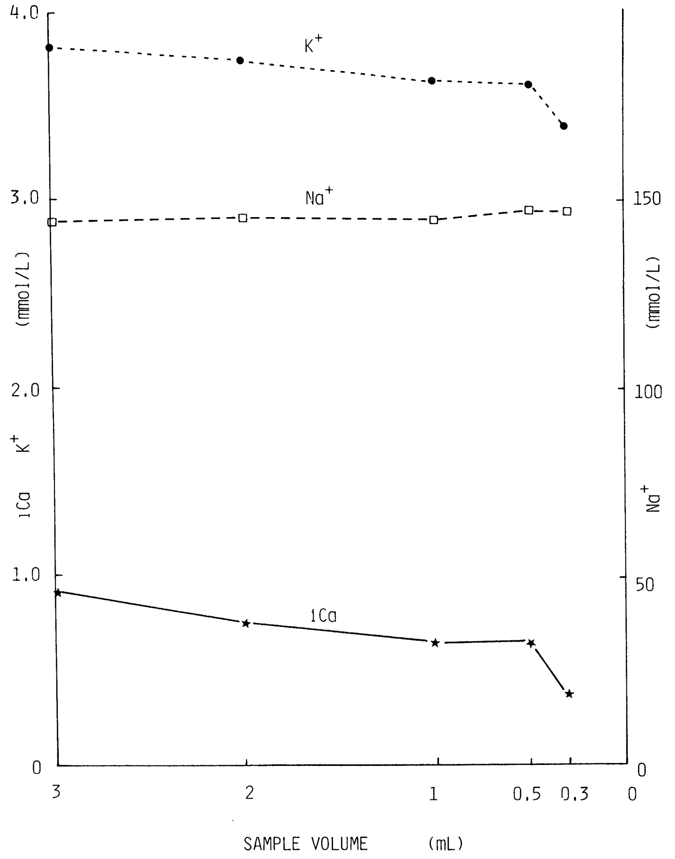

*Figure 3. Varying ratio of sample: anticoagulant. Effect on electrolytes.*

*Note:* When all visible sodium heparin is expelled from the Pulsator syringe, there is still 150 µl remaining in the 'dead space' – i.e. 150 U.S.P. units of heparin sodium remain (Concord Laboratories Ltd).

*Patient samples*

iCa and tCa levels are compared in figure 4 showing relative difference from the reference range for both (identical scale). Student's t-test showed a significant difference ($p$ = <0.05) when values over the upper limit of normal for each reference range were compared.

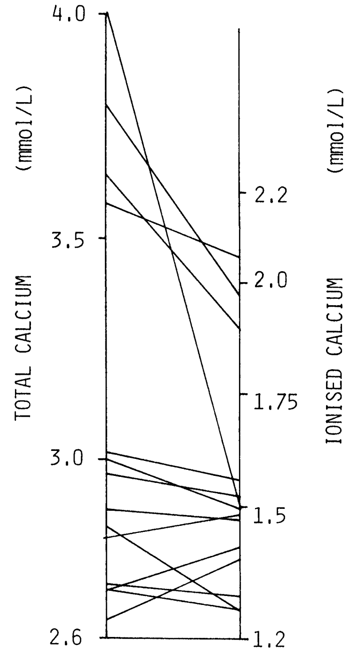

*Figure 4. Total and ionized calcium – varying relation to upper limit of reference ranges in 14 hypercalcaemic patients.*

*Running costs*

From list supplied by UK distributors in January 1987:

Total running costs during the evaluation = £825
Total samples analysed = 1670
i.e. cost per sample = 42p
Basic costs: running the instrument per annum without warranty, for maintenance alone = £1221.06. Calibration costs: varying daily frequency of calibration.

| × 1/24 h | × 3/24 h | × 12/24 h |
|---|---|---|
| £130 | £460 | £1450 |

Eight-hourly calibrations would be required for reasonable precision.
Sample costs: without maintenance of calibration.
Reagent pack for 500 samples = £130–500 = 26p
iCa electrode for 600 samples = £30–600 = 5·8p
Total = 31.8p

Projected cost per sample:

For 5000 samples per annum with 8 hourly calibration:

| | | £ |
|---|---|---|
| Maintenance costs | | 1221·60 |
| Calibration costs | | 460·00 |
| | Total | 1681·60 |
| Divided by number of samples | | ·336 |
| Plus cost per sample (q.v.) | | ·318 |
| | Total = | 65.4p |

For 10 000 samples per annum with 8 hourly calibration:

| | | £ |
|---|---|---|
| Maintenance & calibration costs | | 1681·60 |
| Divided by number of samples | | ·168 |
| Plus cost per sample (q.v.) | | ·318 |
| | Total = | 48·6p |

*User appraisal*

The instrument proved to be robust and reliable, and performed well both in high throughput situations and as a stat analyser. All staff found it easy to use. Maintenance procedures were straightforward and simple, and the instruction manual was clear and easy to follow. The manual supplied with the instrument was comprehensive.

Users found the error codes confusing, in that the difference between major error codes which need operator intervention, and minor error codes, which only indicate a warning signal, but do not require operator intervention, are not clearly differentiated. The flashing light should be reserved for the major error codes only. This would provide a useful indication to the operator as to when actual intervention, rather than merely a general alertness, was required.

### Discussion

The NSPI showed good performance for bias and precision, not only for the manufacturer's control material, where similar or better precision to that claimed by previous workers was recorded (7), but also for tonometered whole blood. Patient sample comparison showed good agreement for pH and blood gases, sodium and potassium measurements. Hct comparison with patient samples showed less good agreement.

During the evaluation, the NSPI had to be moved three times because of departmental changes; handling was carried out each time by the UK agent, and the instrument's performance was unaffected. At the first site, the microprocessor on the NSPI was found to be sensitive to voltage surges occurring from mobile X-ray equipment, necessitating repeated 'set-up' procedures. A voltage stabilizer (spike smoother) was attached to the instrument, and no further problems occurred. The instrument conforms to ESCHLE requirements (Electrical Safety Code for Hospital Laboratory Equipment), and interfaces with RS 232 and Modem.

The cost of using the instrument varies depending on the workload each day, and cost per sample decreases with increased sample throughput. With the option of running fewer autocalibrations, more samples can be run from one reagent pack. Projected costs based on reduced number of daily autocalibrations show a corresponding decrease in cost per sample. The manufacturer is introducing options for reducing the number of autocalibrations per day. Override may be operated on autocalibrations for up to 30 min.

### Ionized calcium

The addition of ionized calcium to the range of tests provided was felt to be a useful adjunct. Many areas of potential use have been listed for iCa measurement. [2 and 10], including the care of the multiply transfused, diagnosis and management of parathyroid disease, in patients with abnormal serum proteins and in liver transplantation. The reliability of the NSP1's iCa electrode, together with the built-in maintenance and performance control from the autocal programme, offer a convenient and reliable method for determining calcium status [8]. This is particularly so when sample handling for iCa analysis in samples other than arterial blood requires some care. [6, 9].

Plasma samples were shown to need centrifuging and analysis within one hour of being taken. Sample bottles should be filled as completely as possible to minimize the effects of both carbon dioxide loss and anticoagulant complexing. Appreciable error (see Figure 3) can be caused by the latter, which also affected potassium measurements.

In the hypercalcaemic patients, it can be seen that there is no consistent relation between the iCa and tCa levels above the upper limit of normal. However, a correlation between serum iCa and albumin concentration similar to that observed by other workers was noted [10].

With the proviso that special precautions should be taken to avoid artefacts which might give rise to the reporting of erroneous iCa results, iCa measurement satisfies a particular need in patient management. A blood gas and electrolyte analyser combines sample requirements, and with its in-built monitoring and calibration facilities offers a reliable and rapid service.

### Acknowledgements

We would like to thank Mike Brown, Haematology, Central Middlesex Hospital, Colin Dickens, Chemical Pathology, Northwick Park Hospital, and Bob Ranger, formerly of Clandon Scientific Ltd, for their help.

### References

1. Siggaard-Andersen, O., Mode, J., Wandrup, J. The concentration of free calcium ions in the blood plasma. 'Ionised Calcium' IFCC, EPpH workshop Copenhagen, 1980.
2. Vanstapel. F. J., Lissens, W. D. Free ionised calcium – a critical survey. *Ann Clin. Biochem*, **21**, 1984, 339–351.
3. Oesch, U., Ammann, D., Simon, W. Ion-Selective Membrane Electrodes for Clinical Use. *Clin. Chem*, **32/8** (1986), 1448–1459.

4. Toffaletti, J., Blosser, N., Kirvan, K. Effects of Storage, Temperature and Time before Centrifugation on Ionized Calcium in Blood Collected in Plain Vacutainer Tubes and Silicone-Separator (SST) Tubes. *Clin. Chem* 1984; **30/4:** 553–556.
5. Deming, W. E. *Statistical Adjustment of Data.* John Wiley & Sons, New York, N.Y. 1943: p. 184.
6. Smith, S. C. H. International ionised calcium meeting – report. *News Sheet Assoc. Clin. Biochem.*, 1986; **278:** 10–12.
7. Johnson, M. T., Savory, J., Wills, M. R. Evaluation of the Nova Stat Profile 1. *Am. Clin. Prod. Rev.*, 1986; Oct.
8. Bowers, G. N. Jr, Brassard, C., Sena, S. F. Measurement of ionised calcium in serum with ion-selective electrodes; a mature technology that can meet the daily service needs. *Clin. Chem.* 1986; **32:** 1437–1447.
9. Burns, J., Law, R. W. G., Paterson, C. R. Measurement of plasma ionised calcium: comparison of calcium-titrated sodium heparin and lithium heparin as anticoagulants. *Med. Lab. Sci.* 1986; **44:** 187–189.
10. Butler, S. J., Payne, R. B., Gunn, I. R., et al. Correlation between serum ionised calcium and albumin concentration in two hospital populations. *Br. Med. J.* 1984; **289:** 248–50.

### Calendar

### FEBRUARY

8–9 February 1989 *Flow Analysis Methods: London, UK.* Contact Analytical Division, RSC, Burlington House, Piccadilly, London W1V 0BN.

### MARCH

6–10 March 1989 *Pittsburgh Conference 1989: Atlanta, GA, USA.* Contact Pittsburgh Conference, Suite 322, 12 Federal Drive, Pittsburgh, PA 15235, USA.

7–9 March 1989 *Workshop: Statistics for Analytical Chemists: Eastbourne, UK.* Contact Christine Robinson, Statistics for Industry (UK) Ltd, 4 Victoria Avenue, Knaresborough, North Yorks HGF 9EU, UK.

21–22 March 1989 *Research and Development Topics in Analytical Chemistry: Dublin, Ireland.* Contact Analytical Division, Royal Society of Chemistry, Burlington House, Piccadilly, London W1V 0BN.

### APRIL

4–7 April 1989 *RSC Annual Congress: Hull, UK.* Contact Gina B. Howlett, Conference Officer, RSC, Burlington House, Piccadilly, London N1V 0BN. The Analytical Division session will be on *Process Analysis and Information Management.*

11–14 April 1989 *VIIth International Symposium on Electroanalysis and Sensors in Biomedical, Environmental and Industrial Sciences: Loughborough, UK.* Contact Dr A. G. Fogg, Chemistry Department, University of Loughborough, Leicestershire LE11 3TU.

### JUNE

25–30 June 1989 *Eurosensors III and 5th International Conference on Sensors and Actuators: Montreux, Switzerland.* Contact Eurosensors (Transducers '89), COMST S.A., PO Box 415, 1001 Lausanne 1, Switzerland.

### JULY

30 July–5 August 1989 *SAC 89: Cambridge, UK.* Contact Analytical Division, Royal Society of Chemistry, Burlington House, Piccadilly, London W1V 0BN.

### SEPTEMBER

11–13 September 1989 *RSC/SCI/IOP Joint International Symposium on Surface Analysis Techniques and Applications: Manchester, UK.* Contact Mrs E. S. Wellingham, Field End House, Bude Close, Nailsea, Bristol BS19 2FQ.

25–27 September 1989 *Sensors and their Applications IV: Canterbury, UK.* The Meetings Officer, Institute of Physics, 47 Belgrave Square, London SW1X 8QX.

25–28 September 1989 *Third International Symposium on Kinetics in Analytical Chemistry: Dubrovnik, Yugoslavia.* Contact Professor Gordana A. Milonović. Department of Chemistry, University of Belgrade, KAC PO Box 550, 11001 Belgrade, Yugoslavia.

26–28 September 1989 *The British Laboratory Week: Olympia, London.* Including Laboratory 89, Computer Aided Sciences, Bio 89, Medical Laboratory Sciences and Analyticon. Contact Curtis Steadman and Partners, The Hub, Emson Close, Saffron Walden, Essex CB10 1HL.

